# Case of Resection of the Head of the Humerus

**Published:** 1868-07

**Authors:** Jno. N. Monmonier

**Affiliations:** Demonstrator of Anatomy Washington University, Balto., Md.


### ARTICLE. VI.

### *Case of Resection of the Head of the Humerus.*

By Jno. N. Monmonier, M.D. Demonstrator of Anatomy Washington University, Balto., Md.

Capt. Henry Charles, Co. A. 5th La. Regt., Hays' Brig.; C. S. A., was wounded, (Sept. 22nd, 1864, at the battle of Fisher's Hill, Va.,) in the right shoulder, the ball (minnie) entering upon the outer surface of the shoulder, fracturing the head of the humerus, the fracture extending several inches into the shaft of the bone, traversing the glenoid cavity of the scapula and emerged through the integument covering the dorsum of the scapula. I saw him a few minutes after the reception of the injury when his nervous system was much shocked. Upon the administration of stimulants, he soon rallied when I proceeded to operate. Chloroform was administered. The operation of resection was performed in this manner. After the patient had been placed in the proper position, a straight incision about three inches in length was made with a scalpel upon the outer surface of the shoulder, the upper portion commencing at a point slightly below the acromion process of the scapula and carried directly down upon the head and the superior portion of the shaft of the humerus. The deltoid muscle having been separated from the outer anterior and posterior surfaces of the bone, the capsular ligament of the shoulder-joint was next divided, then the soft parts upon the inner side of the bone were carefully dissected up, (without wounding a single vessel of much size,) the elbow forcibly brought across the front of the chest and pressed upward and outward, so as to throw the head of the bone, with its fragments, from the glenoid cavity of the scapula. Finally a chain saw was thrown, three inches below the head, around the shaft, when the fractured portions were removed by sawing. In this operation at least four inches of the upper portion of the bone were removed and there was scarcely a table-spoonful of blood lost. It was now near dark and as our forces were flanked and were being rapidly pushed back I had my patient placed in an ambulance and sent him rapidly to the rear. This was the last I saw of him. During the remainder of the war I heard nothing from him as I was soon after this date, transferred to another command.

On the 22nd of April 1868, much to my surprise, I received a letter from him, a portion of which I subjoin.

*Alex. Va. April 20th* 1868.

Dr. Jno. N. Monmonier,

Dear Sir,

You will doubtless be somewhat surprised at receiving a letter from me. But I will have to re-introduce myself. Do you recollect in an old house, at the battle of Fisher's Hill, resecting my right shoulder? Well I am here and am happy to inform you that with your care and the help of God, I am able to write to you this day with that same right arm. I am also happy to inform you that I enjoy tolerable health since I have been employed upon the Orange, Alexandria and Manassa Gap R. Road Very Respectfully,

Henry Charles,

Late Capt. Co. A. 5th La. Regt.

---

### CORRESPONDENCE.

---

